# Active Colloid Phase Transitions and Living Binary Crystal Formation

**DOI:** 10.1021/acsnano.5c19183

**Published:** 2026-02-04

**Authors:** Jingyuan Chen, Shaobin Zhuo, Binglin Zeng, Zhigang Li, Jinyao Tang

**Affiliations:** † Department of Chemistry, 25809 The University of Hong Kong, Pokfulam 999077, Hong Kong, China; ‡ Department of Mechanical and Aerospace Engineering, 121835 the Hong Kong University of Science and Technology, Clear Water Bay, Kowloon 999076, Hong Kong, China; § State Key Laboratory of Synthetic Chemistry, The University of Hong Kong, Kowloon 999076, Hong Kong, China; ∥ Materials Innovation Institute for Life Sciences and Energy (MILES), HKU-SIRI, Shenzhen 518000, China

**Keywords:** photoactive colloids, colloidal interaction, phase transition, Langevin dynamics, binary crystal

## Abstract

Colloids can be utilized as model “meta-atoms” to emulate phase behaviors at the atomic scale for easy observation and slower dynamics. Photoactive colloids have recently been demonstrated with on-demand directional interactions as well as tunable dynamics, which are particularly suitable to emulate the phase transition of atomic lattices due to their excellent tunability. In this study, we demonstrate that the photochemical reaction on active colloids can induce an optically tunable hydrodynamic interaction field. By spontaneously controlling the directional interaction and omnidirectional repulsion with two sets of illumination, the phase transition between the zigzag band, chains, and dispersed phase, distinguished by their 2-fold bond orientational order, can be realized. Furthermore, the addition of passive colloids, analogous to reactant atoms with different chemical natures and sizes, causes a “chemical reaction” between the colloid species, forming colloid compounds with well-defined stoichiometric ratios, while the phase transition of the colloid compound can also be emulated with external illumination. By bridging active matter physics and solid-state chemistry, our platform provides a versatile tool for studying phase diagrams and optically encoding “reaction pathways” in colloidal alloys.

## Graphical abstract

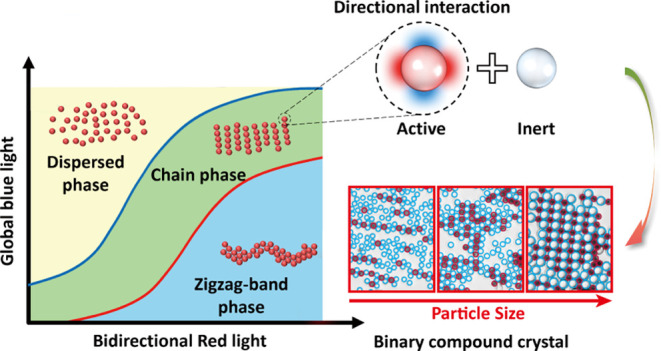

Colloids are well-known for their capability of forming various assembly structures and patterns under either thermal equilibrium or with energy dissipation.^1–3^ The tunable surface properties, pairwise interactions, and similarity to atomic kinetics enable colloids to emulate phase transitions at the experimentally more accessible spatial and temporal resolution.^4,5^ For instance, the particle–particle interaction can be fine-tuned by implementing temperature-sensitive coatings, polymeric additives, and dye molecules, where the resulting phase transition dynamics and detailed mechanisms can be studied.^6–10^ Active colloids, which generate mechanical force by dissipating energy, render additional degrees of freedom to emulate the atomic-scale phase transitions with versatile tunability, including optical, electric, magnetic, and chemical stimulation.^1,11^ In particular, photoactive colloids generate the hydrodynamic interaction via surface photochemical reaction, demonstrating outstanding versatility and tunability.^12^ Many dynamic assemblies like living crystals, vortices, flocking, swarming, and synchronizing assemblies have been previously realized in photoactive colloids.^13–17^ Specifically, for spherical or highly symmetric active colloids, which generate the peripheral flow without net motion, an apparent potential may describe the particle–particle interaction semiquantitatively and result in the corresponding phase behavior.^14,18^ This unique feature enables the photoactive colloids to serve as a powerful model to emulate the phase behaviors of materials at equilibrium with faster dynamics or even inaccessible phase transitions.

Previously, we showcased that the directional interaction could be created by tuning the illumination intensities and directions on photoactive colloids, where five Bravais lattices can be reversibly induced,^18^ indicating the ability of photoactive colloids as tunable surrogates for different atoms. Here, we demonstrate the versatility of the photoactive colloid system as a physical model to emulate phase transitions between zigzag bands, nematic chains, and the dispersed phase. By adding another colloidal species into the system, “chemical reactions” occur between two colloid species, forming binary compound crystals and compound polymers with different stoichiometries, where the phase transition can be controlled and studied on demand. This approach bridges equilibrium and active matter physics and unlocks unprecedented control over phase behavior in synthetic systems, offering an intuitive approach to understanding colloid self-organization.

## Results and Discussion

### Characterization of Particle Interaction

In this experiment, monodispersed TiO_2_ microspheres (Figure S1) with a diameter of 2.5 μm were sensitized with LEG4 dye sensitizer and dispersed in 0.1 M ferrocene solution in acetonitrile. This colloid solution was then wax-sealed in a glass capillary that served as the experimental chamber. The experimental setup is shown in Figure 1a; horizontal bidirectional red light (660 nm, *I*_R_) and vertical blue light (460 nm, *I*_B_) are spontaneously illuminated on the sample, where red light generates bipolar attractive force along the illumination direction and blue light generates omnidirectional repulsive force.^18^ Notably, the collimated LEDs in the bidirectional red-light illumination are not perfectly horizontal (slightly tilted with an angle of 10°), which eliminates the light attenuation between particles.

**Figure 1:**
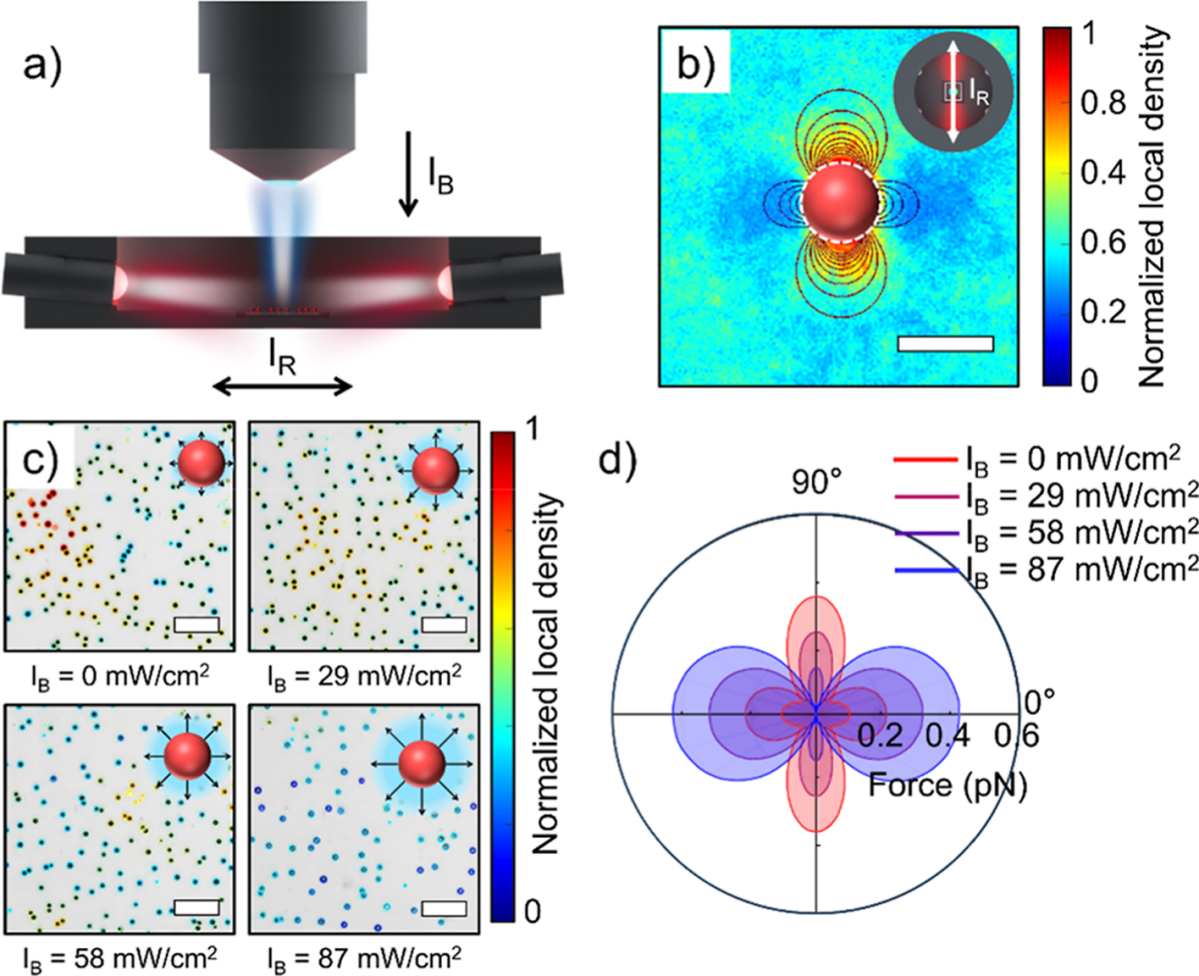
Experimental setup and particle behavior under illumination. (a) Schematic of the horizontal bidirectional red-light illumination (*I*_R_) and blue-light illumination from top (*I*_B_). (b) Top view of the normalized local density of the 500 nm SiO_2_ tracer particles around a fixed LEG4-TiO_2_ active particle under *I*_R_ = 59 mW/cm^2^, showing an orthogonally arranged repulsion/attraction field. The bright arrow in the inset indicates the illumination direction. (c) Distribution of active particles under blue-light illumination with different intensities. Particles are colored by their local number density within the range of 5 particle diameters. (d) Simulated force curves around the active particle under *I*_R_ = 59 mW/cm^2^ and *I*_B_ = 0, 29, 58, 87 mW/cm^2^. Scale bars: (b) 3 μm and (c) 20 μm.

To visualize the colloid interaction under bidirectional red-light illumination, we first injected a dilute LEG4-TiO_2_ suspension into a capillary and dried it to fix the LEG4-TiO_2_ particles on the substrate. Subsequently, a solution containing 500 nm silica tracers and 0.1 M ferrocene was added, and the solution was wax-sealed. Upon horizontal bidirectional red-light illumination, the photonic nanojet effect leads to higher light intensities at the focal point of the active particles, boosting the redox reaction that generates an orthogonally arranged hydrodynamic flow field. To elaborate, the LEG4 dye sensitizer primarily absorbs blue light but also exhibits absorption in the red-light region.^19,20^ When LEG4-sensitized TiO_2_ particles are exposed to red or blue light, electron–hole pairs are generated and separate on the particle surface. The holes oxidize ferrocene into ferrocenium ions, while the electrons reduce ferrocenium ions back to ferrocene, where the ferrocene/ferrocenium couple acts as a redox shuttle.^21^ Owing to the porous structure of the synthesized TiO_2_ particles, the microcrystals on the surface function as numerous microscopic anodes and cathodes. This results in a net directional diffusion of cations and anions, and the difference in their diffusion rates establishes a self-generated electric field that ultimately drives the particle motion. Regarding the focusing effect observed in the TiO_2_ particles, our previous findings indicate that it arises from their distinct optical behaviors under blue and red light.^18^ While the particles are essentially opaque to blue light, they are semitransparent to red light, thereby acting as spherical lenses that focus red light to their focal points. This leads to enhanced localized light intensity and, consequently, stronger light absorption and photoredox reactions in that region.

As shown in Figure 1b, the high-density tracer zone is observed along the illumination axis, while the low-density zone shows repulsion, forming a p-orbital-like potential around the fixed LEG4-TiO_2_ particle. On the other hand, when the active particles are illuminated with only blue light from the top, they can generate omnidirectional repulsion. The photoactive particle distribution under different blue-light illumination is plotted in Figure 1c and Movie S1, where the color indicates the local particle densities within the range of 5 particle diameters. Stronger blue-light illumination enhances the strength of the interparticle repulsion, causing the decay of particle density and the formation of a dispersed state.

Moreover, our previous study shows that the blue-light illumination can modify the overall shape of the hydrodynamic flow field around the active particles.^18^ By combining the previously described method and data to extract the relationship between light intensities and reaction flux, we calculated and plotted the force field around an active particle using COMSOL simulation under four blue light intensities (Figure 1d and Figure S2), revealing a decrease of attraction strength along the bidirectional illumination direction and an increase in repulsion strength in the perpendicular direction.

### Phase Transition of the Photoactive Colloids

To study the phase behavior under different illumination conditions, we first investigated the growth behavior of the active particles under bidirectional red-light illumination. With an area fraction of 30% and *I*_R_ = 59 mW/cm^2^, particles initially form short chains along the illumination direction and then rapidly collapse into horizontal zigzag band-like patterns, with particles circulating along the edges of the bands. Figure 2a shows the snapshots of the zigzag dynamic pattern formation within the first 30 s. These bands continue to grow until they reach a stationary state where several large bands coexist in different areas, which can be defined as the “zigzag band” phase.

**Figure 2:**
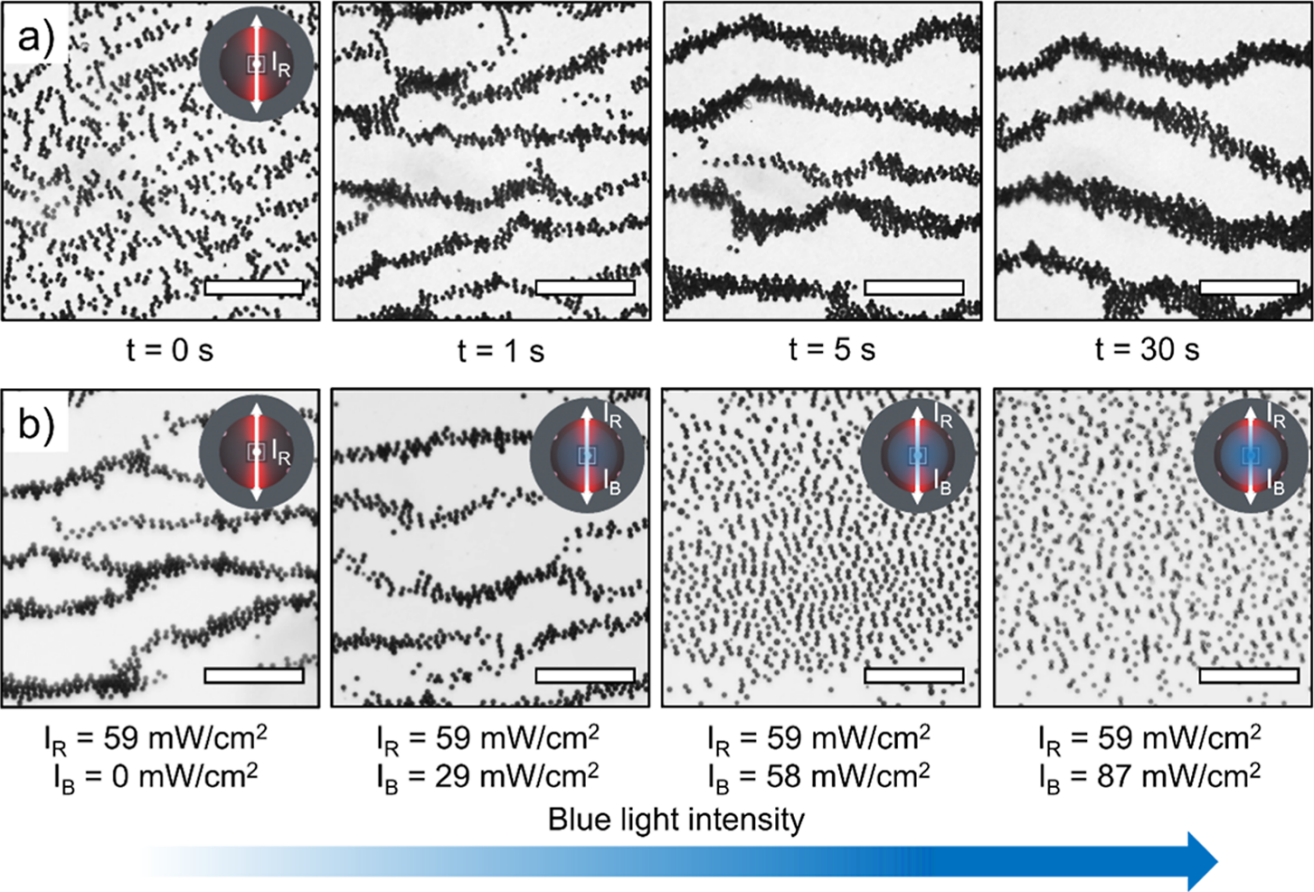
Formation of zigzag bands and the active phase transition. (a) At an area fraction of 30% and *I*_R_ = 59 mW/cm^2^, active particles form zigzag bands upon illumination, and the bands keep growing within the first 30 s into larger bands. (b) Phase transition between the zigzag band phase, chain phase, and dispersed phase upon increasing *I*_B_. Scale bars in (a, b): 100 μm.

After using red light to initiate the zigzag band phase, we utilized the aforementioned blue-light illumination *I*_B_ to gradually apply the repulsion between the particles. As an analogy of solid melting, higher blue light intensity will lead to more dispersed states with higher system entropy. As shown in Figure 2b and Movie S2, at low blue-light illumination (*I*_B_ = 0–29 mW/cm^2^), the colloidal particles form a zigzag band with locally high particle density. By increasing the repulsion with illumination, the active fluctuation and repulsion increased simultaneously, so that the zigzag bands transformed into colloid chains. With even stronger illumination, the fluctuation dissociated the dense colloid chains into a highly dispersed state similar to that in Figure 1c.

In order to study the structural evolution during this phase transition, we utilized the bond orientational order parameter ψ_*m*_^*j*^ to characterize our colloid system, which has been employed to study phase transitions of crystal melting (*m* = 6)^22,23^ and quasicrystallization (*m* = 12).^24^ In our system, since the bidirectional illumination direction defines the orientation of colloidal assembly, we use the 2-fold (*m* = 2) bond orientational order parameter $\psi_{2}^{j}=\sum_{k=1}^{N_{j}}e^{2i\theta_{k}^{j}}/N_{j}$ , which shows the nematic order of the assembly structure, where θ_*k*_^*j*^ is the angle of the bond between particle *j* and its neighbor *k* and *N*_j_ is the number of nearest neighbors. Figure 3a and Movie S3 show the particle assemblies with various blue light intensities, colored by their orientational order parameter (ψ_2_^*j*^). Under low light intensities, the colloid forms alternating zigzag band structures without nematic order. As the light intensity increases to 58 mW/cm^2^, the nematic order suddenly emerges and forms long colloid chains. Further increasing the blue-light illumination breaks down the colloid chains into single particles and short chains, lowering the nematic order again.

**Figure 3:**
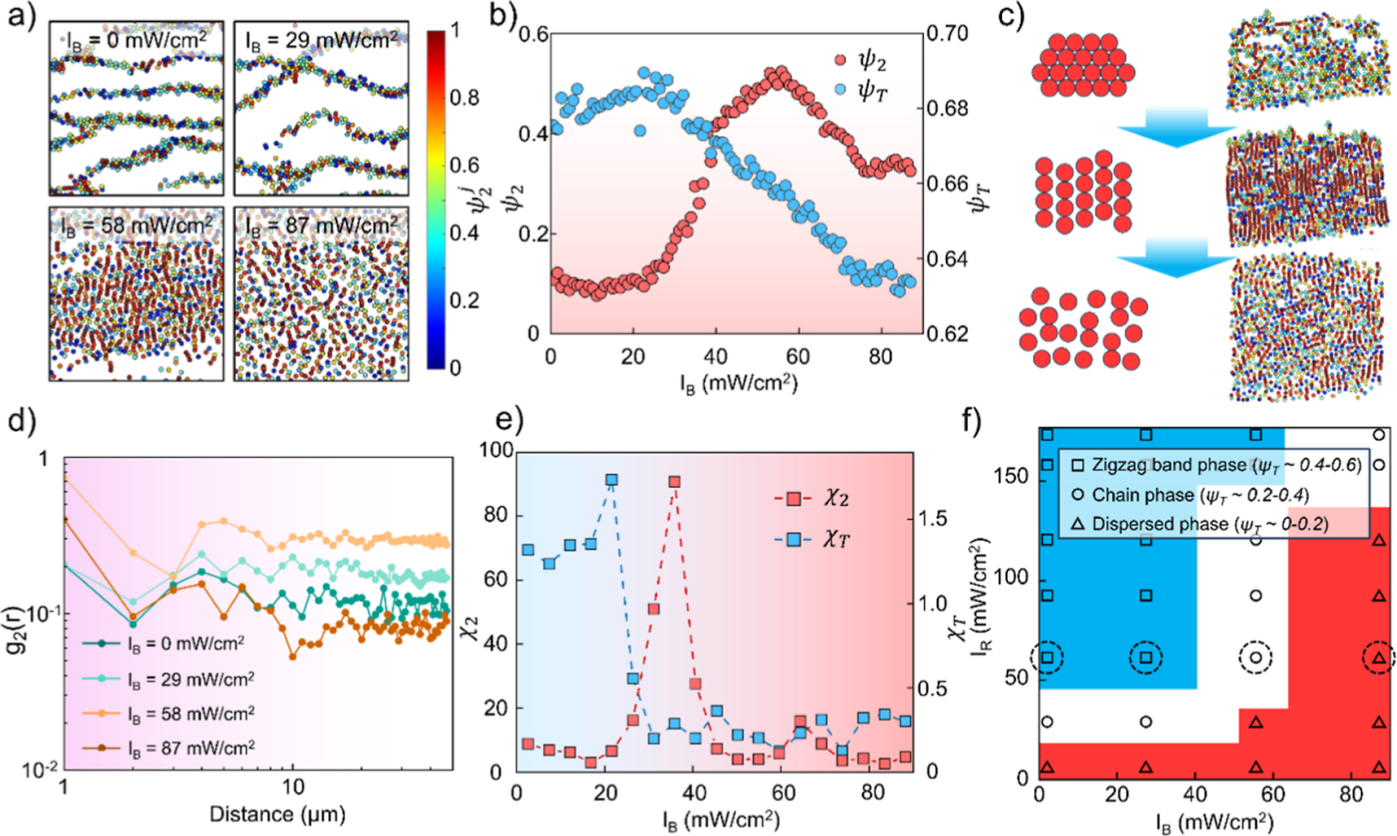
Structural analysis and phase behavior of photoactive colloids. (a) Particle assemblies colored by their bond orientational order parameter ψ_2_^j^ under four different *I*_B_, indicating the emergence of nematic order. (b) Total bond order parameter ψ_2_ and translational order parameter ψ_T_ change upon increasing *I*_B_, showing a continuous phase transition. (c) Phase transition process of the active colloid system from crystal chains to the dispersed phase. (d) The bond orientational order correlation function *g*_2_(*r*) under four different *I*_B_ corresponding to 2-fold symmetry at a distance r from a reference particle. (e) Translational and orientational susceptibilities upon increasing *I*_B_. Experiments in (a)–(e) were all conducted under *I*_R_ = 59 mW/cm^2^. (f) Experimental phase diagram under different red light intensities *I*_R_ and blue light intensities *I*_B_. Different phases can be roughly distinguished by their averaged ψ_T_. The circled data represent the experimental results in Figure 3a.

We plotted and compared the evolution of bond orientational order and translational order upon increasing blue light intensity, as shown in Figure 3b. The definition of the translational order parameter ( $\psi_{T}^{j}=e^{iG\cdot r_{j}}$ ) is adopted from previous literature,^22^ where *G* is a primary reciprocal lattice vector determined from the peak of the 2D structure factor *s*(*k*) at each blue light intensity. As *I*_B_ increases, the total translational order parameter ψ_T_ decreases due to the increase in both interparticle repulsion and the particles’ increasing active fluctuations, while for the total bond orientational order parameter ψ_2_, the nematic order quickly rises as the repulsion increases and reaches its maximum at *I*_B_ = 58 mW/cm^2^. Higher repulsion force also disturbs the bond orientational order by breaking the chains down into shorter chains, but the overall nematic order is still higher than that of the initial crystal structure. This result shows a very different “melting” route compared to the 2-dimensional crystal melting under thermal equilibrium suggested by the Kosterlitz–Thouless–Halperin–Nelson–Young (KTHNY) theory^25–27^ in two ways. (1) In our photoactive colloid system, the particle interactions are symmetry-broken, leading to a nematic order where the active particles tend to align with the external bidirectional red light field. (2) The blue-light illumination not only provides active fluctuation but also modifies the shape of the interaction force field (Figure 1d), generating long-range repulsion perpendicular to the bidirectional illumination direction that leads to “directional” dispersion. Therefore, as shown in the “melting” process of the active colloids in Figure 3c and Movie S4, instead of the formation of the intermediate hexatic phase, the colloids form an intermediate phase with nematic order, where particles form aligned long chains with uniform chain-to-chain distances around 1.5 particle diameters. The bond orientational order correlation function *g*_2_(*r*) is constructed with ψ_2_^j^, measuring the correlation of bond orientations with respect to 2-fold symmetry at a distance r from a reference particle. Figure 3d shows the emergence of quasi-long-range orientational order as light intensity increases to 58 mW/cm^2^, and it vanishes under higher *I*_B_.

To determine the critical point of phase transition, we calculated the order parameter susceptibility χ, which describes the order parameter fluctuations, where the orientational susceptibility (χ_2_) and translational susceptibility (χ_T_) are defined as χ_2_ = *L* ^2^(⟨|ψ_2_|^2^⟩ – ⟨|ψ|⟩^2^) and χ_T_ = *L* ^2^(⟨|ψ_T_|^2^⟩ – ⟨|ψ|⟩^2^), respectively. Here, *L* is the system size, and ψ_2_ and ψ_T_ are the mean order parameters over all *N* particles in the *L* × *L* box. As shown in Figure 3e, the susceptibility curves diverge at *I*_B_ = 21 mW/cm^2^ and 36 mW/cm^2^ for χ_T_ and χ_2_, respectively. This critical point discrepancy indicates that the translational order vanishes prior to the orientational order, corresponding to the sequential disappearance of the crystal structure and emergence of the nematic structure. Notably, the orientational susceptibility curve shows a secondary peak at *I*_B_ = 64 mW/cm^2^, which refers to the breakdown of the chain-like structures under increasing repulsion and active fluctuation.

Another key factor that affects the phase stability is the interparticle bonding strength, which can be modulated by the bidirectional red-light intensity *I*_R_. The resulting colloid assembly is more robust with a stronger bonding strength (higher *I*_R_), allowing the structure to withstand higher blue light intensity *I*_B_. By tuning different combinations of *I*_R_ and *I*_B_, the transition between the zigzag bands, chains, and dispersed phase can be induced on-demand, and an experimental phase diagram is generated accordingly, distinguished by their ψ_T_ values (Figure 3f). Notably, the “zigzag bands” were observed under a relatively low particle density with the bidirectional illumination setup similar to our previous work. To illustrate the difference, we tested and summarized the experimental results with different initial areal densities in Figure S3. Higher initial density results in larger bands and more randomly dispersed band widths. On the one hand, concentrated particles generate a high quality of chain-like colloidal crystals, as shown in the last panel in Figure S3c. This particle density benefits the following polymorphic colloidal crystal assembly in our previous paper. On the other hand, an areal density around 30% provides the clearest zigzag band structures (second and third panels of Figure S3b), allowing the study of both the dynamics and growth kinetics of the assembly. To summarize, the phases formed by the LEG4-TiO_2_ particles arise within a wide range of areal particle densities (15%–63%), and the choice of density depends on the phase that we are interested in.

### Comparison with Langevin Dynamics Modeling

We hypothesize that the phase transition behavior is determined by the interparticle interactions, which are generated by external light illumination. Although this interaction is intrinsically hydrodynamic and many-body, we argue that a simplified pairwise interaction model can largely capture the assembly behaviors of our experiments. To verify this, we conduct simulations using a custom implementation of Langevin dynamics in C++. In brief, the model simulates particle interactions using a directional potential field corresponding to directional attraction induced by red-light illumination, which is then modified into different orthogonal repulsion/attraction combinations. Due to the varying shape of the interaction field, the nonspherical symmetry of the potential results in the corresponding angular distribution of particles.

The COMSOL simulation is applied first to model the hydrodynamic flow generated by the self-diffusiophoresis of the active particle, which is utilized to determine the overall force field for each “atom” (Materials and Methods) as shown in the inset of Figure 4a. To bridge the complex, nonconservative hydrodynamic interactions with a tractable simulation model, we construct an effective apparent potential based on the anisotropic flow field derived from COMSOL simulations.^14,18,28^ This approach aims to capture the essential features of the interparticle forces, specifically the anisotropic components of attraction and repulsion, rather than to precisely solve the full hydrodynamic equations. Notably, similar phenomena have been reported in alternating electric and magnetic fields, where the colloidal particles can form circulating zigzag bands.^29–32^ The formation of such structures can be attributed to electric dipole-like interaction; therefore, the Yukawa potential (screened Coulomb potential)^33,34^ is adopted as a well-established functional form in colloidal science to model such effective, screened interactions:

$$U_{Yukawa}=\varepsilon_{0}\left(C\frac{e^{-\alpha r}}{\alpha r}\right)$$

where *ε*_0_ is the binding energy, 1/α is the effective range of the screened Coulomb interaction, and *C* is a constant.

**Figure 4:**
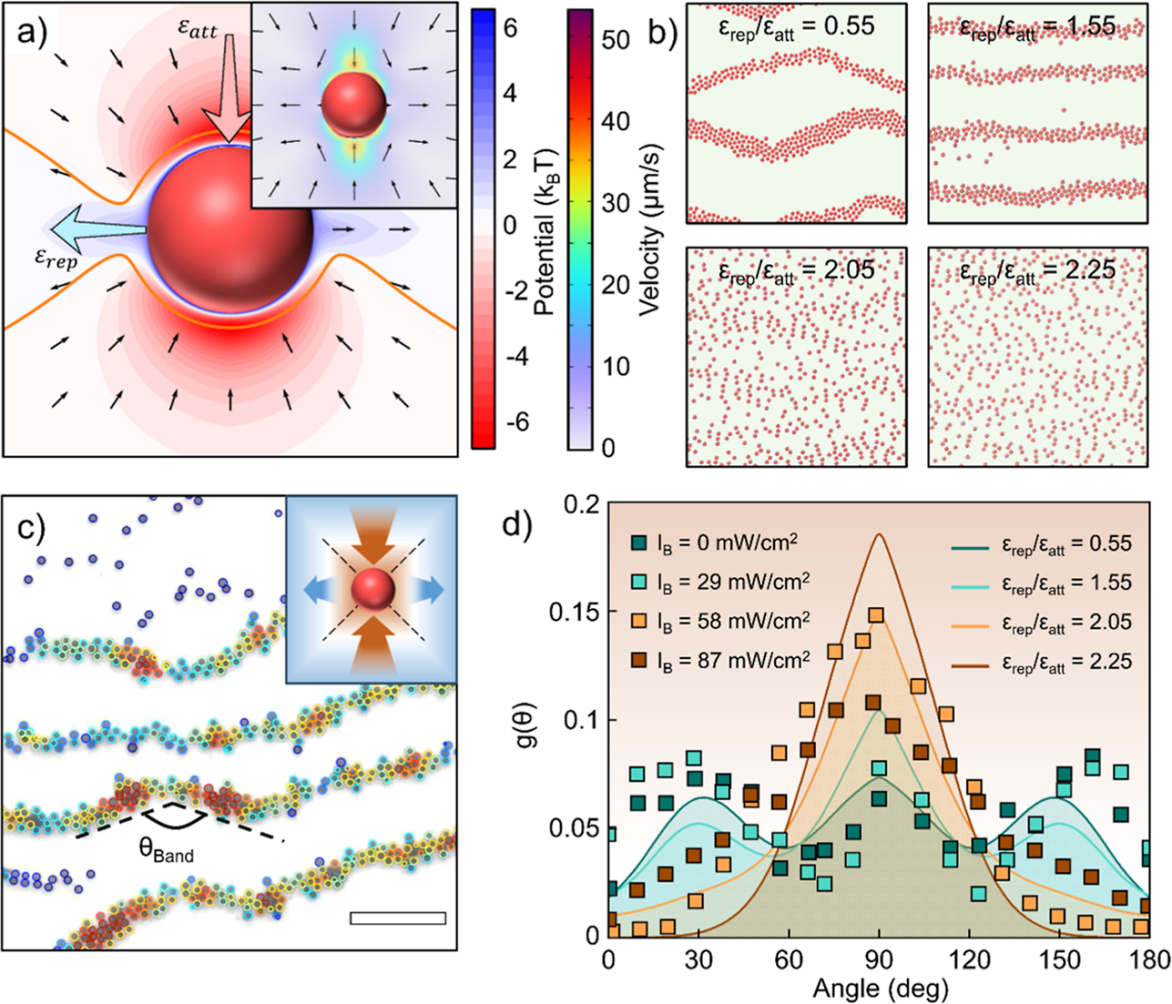
Langevin dynamics simulation and the angular distribution around the active particle. (a) Directional interaction potential constructed based on the COMSOL simulation of hydrodynamic flow (inset). Attraction energy and repulsion energy are denoted as *ε*_att_ and *ε*_rep_, respectively. Dark arrows indicate the force vectors, and the orange lines are stagnation lines where the force equals 0. (b) Langevin dynamics simulation under four different ε_rep_/ε_att_ ratios, replicating the active phase transition. (c) Zigzag band pattern formed at *I*_B_ = 58 mW/cm^2^. Particles are colored by their local number density. *θ*_band_ is the angle of the zigzag bands. The inset shows the stagnation line around the active particle that leads to the formation of the zigzag pattern. (d) Angular distribution function *g*(*θ*) from both experiments (data points) and simulations (lines). Scale bar: 30 μm.

Its anisotropy is introduced through an angular modulation function to replicate the orthogonally arranged force field observed in experiments (Figure 1b) and simulations (Figure 1d, inset). To ensure the formulated potential is derivative and prevent the particles from overlapping in the attractive region, the 2-dimensional potential can be formulated by combining the Lennard-Jones potential with the Yukawa potential,

$$U\left(r,\theta \right)=\left(U_{LJ}-U_{Yukawa1}\right)\sin\;\theta +U_{Yukawa2}\cos\;\theta =\varepsilon_{att}\left(4\left[{\left(\frac{\sigma }{r}\right)}^{12}-{\left(\frac{\sigma }{r}\right)}^{6}\right]-C\frac{e^{-\alpha r}}{\alpha r}\right)\left|\sin\;\theta \right|+\varepsilon_{rep}\left(C\frac{e^{-\alpha r}}{\alpha r}\right)\left|\cos\;\theta \right|$$

where *U*_Yukawa1_ models the directional attraction introduced by red light (*I*_R_), *U*_Yukawa2_ models the omnidirectional repulsion introduced by blue light (*I*_B_), and *σ* is the collision diameter. The region where attractive or repulsive forces are present is determined by a sinusoidal function such that when two particles are arranged tip-to-tip (*θ* ≅ 90° or 270°), they experience attraction, whereas side-by-side (*θ* ≅ 0° or 180°) positioning results in repulsion. Figure 4a shows the constructed directional potential, where the black arrow denotes the force vector surrounding the particle. Notably, the potential does not aim to reproduce hydrodynamic flow but rather serves as an effective interaction model that captures the net result of light-induced forces and hydrodynamic flows observed experimentally. Thus, the Yukawa potential is not a literal fit to hydrodynamic interactions but an effective representation of net interactions, justified by the directional symmetry breaking imposed by the illumination geometry.

At four different repulsion/attraction ratios, the simulation results well replicate the transition from zigzag bands to short chains to the dispersed phase under the directional potential (Figure 4b and Movie S5), indicating that repulsion is sufficient to induce the observed phase transition in this balanced active particle system. It is worth noting that for the first phase, the simulation also reproduces the circulating behavior, where all the particles form zigzag bands and circulate on the edges, showing behavior similar to dipolar interacting colloids.^30,31^ By applying different attraction energy *ε*_att_ and repulsion energy *ε*_rep_, the simulated phase diagram (Figure S4) well matches the experimental phase diagram in Figure 3a.

The simulation allows us to understand the formation and modulation of the zigzag band, particularly the bending angle *θ* between segments of bands as experimentally observed in Figure 4c. To better visualize the cusps of the zigzag bands, the particles are colored by their local particle density within the range of 10 particle diameters. When exposed to bidirectional red-light illumination, the active particle is subjected to an orthogonally arranged repulsion/attraction force created by the directional hydrodynamic flow, where the repulsion/attraction regions balance on two stagnation lines with an angle of 135° (inset of Figure 4c). At low blue light intensities, when two particles meet, they first approach from the direction of attraction, then slide along the stagnation line, and finally separate (Figure S5). This sliding motion affects the overall orientational distribution of the colloids. To determine the system’s angular distribution function *g*(*θ*), each particle is connected to its neighbors, and the probability distribution of the bond angles is counted (Figure S6).

Figure 4d shows the angular distribution functions (*g*(*θ*)) of colloids under varying blue light intensities, as summarized from experiments (data points) and calculated from Langevin dynamics simulation (lines). At low blue light intensity (*I*_B_), three distinct peaks at about 20°, 90°, and 160° can be observed for *g*(*θ*), corresponding to the zigzag band phase. When the *I*_B_ is raised, the three peaks of *g*(*θ*) merge into a single peak at 90°, which corresponds to the potential field contour shaping with light. The repulsion region of the potential field expands while the attraction region shrinks during increasing *I*_B_, resulting in the shift of stagnation lines and thus the narrowing of the probability distribution toward 90°. Nonetheless, at the high repulsion region (*I*_B_ = 87 mW/cm^2^ and *ε*_rep_/*ε*_att_ = 2.25), the experimental *g*(*θ*) and the values predicted by the simulation show a relatively large deviation. This suggests that the enhanced active fluctuation of the particles may also play a role in the particle distribution, which tends to flatten the distribution, causing deviation from simulation results.

### Colloidal Compound Crystal Assembly

In materials science, binary alloys’ phase separation and crystallization are governed by free energy landscapes, component ratios, and kinetic constraints.^35^ Similarly, colloids may assemble under thermal equilibrium, where various phases can emerge by tuning the pairwise interaction potentials.^36–38^ However, the traditional colloidal assemblies are thermodynamically static, as determined by the free energy minimum, lacking the flexibility for in situ switching and programming.^1^ Recent studies on the assembly of living colloidal crystals with active particles demonstrated outstanding spatiotemporal control over the assembly structure and dynamics,^14,18,39,40^ but the assembly of living binary crystals with well-defined stoichiometric ratios has yet to be achieved. With complete control of colloid interactions, our system provides a feasible approach to studying the “chemical reaction” between different colloid species and the formation of colloid alloy crystals.

In order to emulate the process of alloy formation with different atom sizes, we selected passive silica particles with different sizes to be alloyed with our photoactive colloids. During the experiment, 2.5 μm LEG4-TiO_2_ particles are mixed with 2 and 3 μm SiO_2_ particles at molar ratios of 1:2 and 1:1, respectively. As shown in Movie S6, the regular colloid compound crystal is formed with well-defined particle interactions between passive and active colloids. Upon red-light illumination, the active particles attract one or two passive particles at one end first, forming self-propelling active assemblies, which swim away from the passive particle end (Figure S7). This swimming active assembly absorbs other passive particles, forming a balanced structure with one active particle surrounded by four passive particles, corresponding to the stagnation lines in Figure 4a.

As expected, the resulting colloid compound structure varies with relative particle size.^41,42^ A well-ordered binary crystal lattice with a 1:1 stoichiometric ratio was formed with 3 μm SiO_2_ and 2.5 μm TiO_2_ particles (Figure 5a), while a loose chain-like colloid compound polymer with a 2:1 stoichiometric ratio was formed with 2 μm SiO_2_ and 2.5 μm TiO_2_ particles (Figure 5b). This size dependence may be rationalized by the relative repulsion/attraction region size. As large passive particles surround the active particle, its repulsive region in the horizontal direction will be blocked by the large size of the passive particles, making the assembly’s repulsive force less significant in the horizontal direction, leading to the formation of a 1:1 ratio compound crystal. On the contrary, with the active particle surrounded by small passive particles, its repulsive force in the horizontal direction overwhelms the attraction, leading to the formation of stable 2:1 ratio compound chains (Figure S7c-d).

**Figure 5:**
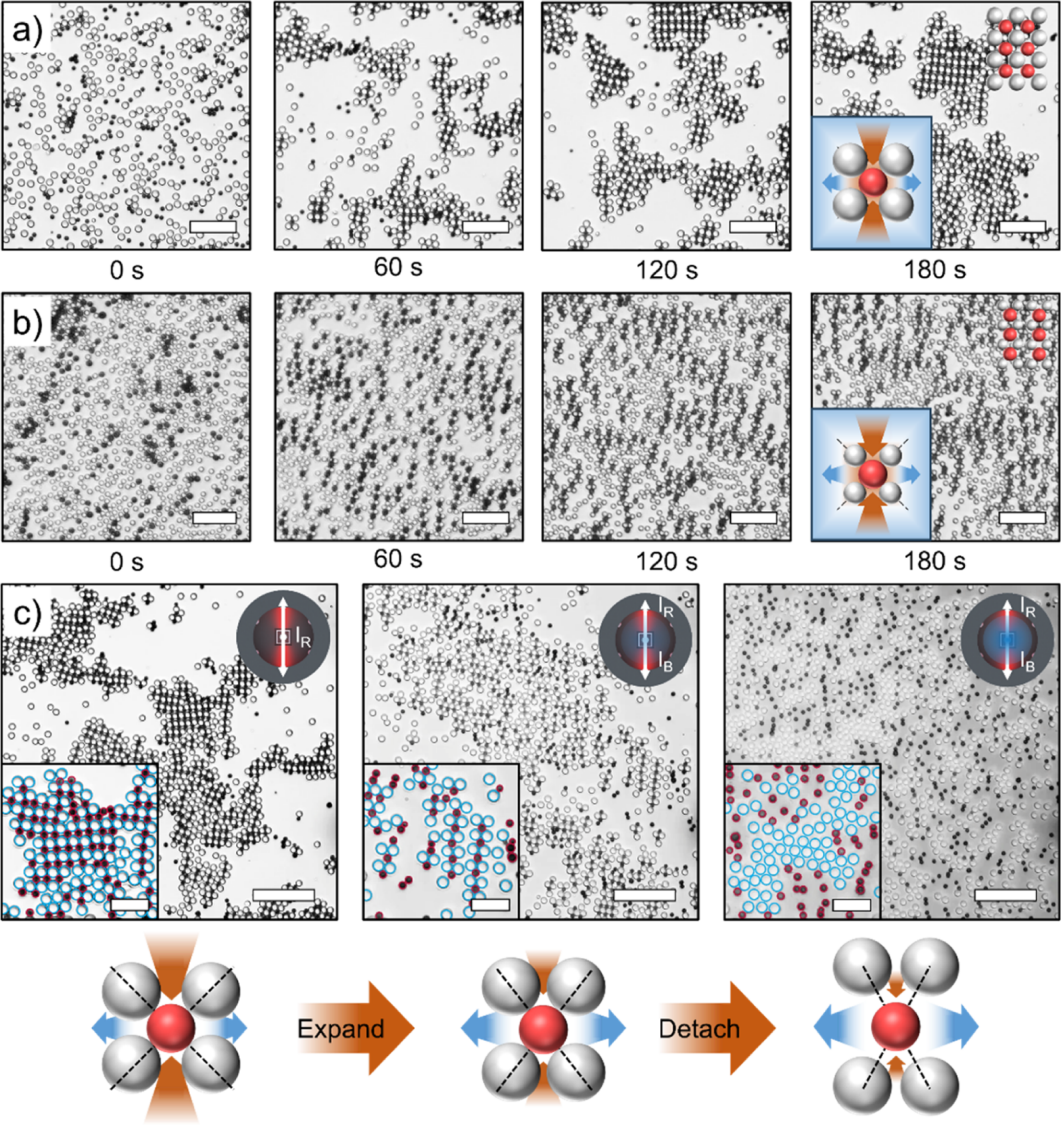
Binary crystal formation between LEG4-TiO_2_ active particles and SiO_2_ passive particles. (a,b) Snapshots of the binary colloidal crystal formation where the TiO_2_ particle is darker than the SiO_2_ particle in microscope images. (a) Binary compound crystal with 2.5 μm LEG4-TiO_2_ particles and 3 μm SiO_2_ particles. (b) Binary compound chains with 2.5 μm LEG4-TiO_2_ particles and 2 μm SiO_2_. (c) Binary crystal phase transition with increasing blue-light illumination and finally reaching phase segregation states. Insets are the magnified images of the corresponding microscope images with TiO_2_ and SiO_2_ highlighted with red and blue circles, respectively. Scale bar: (a,b) 20 μm, (c) 30 μm, and 10 μm (inset).

As an active system, the phase behavior of this binary system can be effectively controlled with illumination. As shown in Figure 5c and Movie S7, the phase transition process of the binary compound crystals undergoes a two-stage transformation under increasing blue-light illumination. First, as the repulsion of the active particles increases, the intersecting angle between the two stagnation lines diminishes, as described in Figure 4d. The lattice of the binary crystals gradually expands and then transforms into an unstable structure similar to the compound chains in Figure 5b at *I*_B_ = 58 mW/cm^2^. When the blue light intensity further increases, the attraction region becomes insignificant compared with the repulsion region of the active particles, causing the SiO_2_ particles to detach. All compound chains are disrupted thereafter, and a dispersed state is observed. Under these conditions, weak TiO_2_-TiO_2_ attraction combined with TiO_2_-SiO_2_ repulsion leads to partial phase separation, arising from the distinct responses of TiO_2_ and SiO_2_ to blue-light illumination. Notably, some close-packed SiO_2_ particle clusters can be observed under intense blue-light illumination (right of Figure 5c). This is due to the long-range repulsive force exerted by the active particles upon blue-light illumination, which causes each photoactive particle to occupy a larger area. This effect enhances the effective volume exclusion and forces the passive particles to occupy the rest of the spatial area, resembling a liquid–liquid phase separation.^43^

Finally, we investigated the dependence of binary phase formation on the relative particle size. Here, we used 2.5 μm TiO_2_ particles as photoactive component, and co-assembled them with passive SiO_2_ particles of varying sizes. It is found that silica particles larger than 5 μm or smaller than 2 μm only form disordered mixtures with TiO_2_ without any well-defined compound lattice. We therefore selected passive SiO_2_ particles with diameters of 2 μm, 2.5 μm, 3 μm, and 5 μm, varying the passive/active particle size ratio from 0.8 to 2, and studied the binary phase evolution under varying blue light intensities. Figure 6 shows the phase diagram with the corresponding microscopic images. For a passive/active particle size ratio of 0.8 (2 μm silica particles), active particles exclusively assemble into compound chains that undergo direct phase segregation upon higher repulsion as imposed by blue-light illumination. Increasing the passive/active particle size ratio (2.5 and 3 μm silica particles) enables the formation of the compound crystal with a 1:1 ratio. Within this particle size ratio region, the phase transition from compound crystal to compound chains and finally to segregated dispersed states can be achieved by increasing the repulsion with blue-light illumination. Further increasing the particle size ratio to 2 (5 μm silica particles) prevents stable compound chain formation as the repulsion region created by the TiO_2_ cannot extend beyond the assembled large silica particles; thus, the system collapses directly from binary crystal into phase-segregated domains upon increasing repulsion, precluding observable compound chain intermediates. Langevin dynamics simulations reproduce these experimental trends (Figure S8), confirming our interpretation of the mechanism.

**Figure 6:**
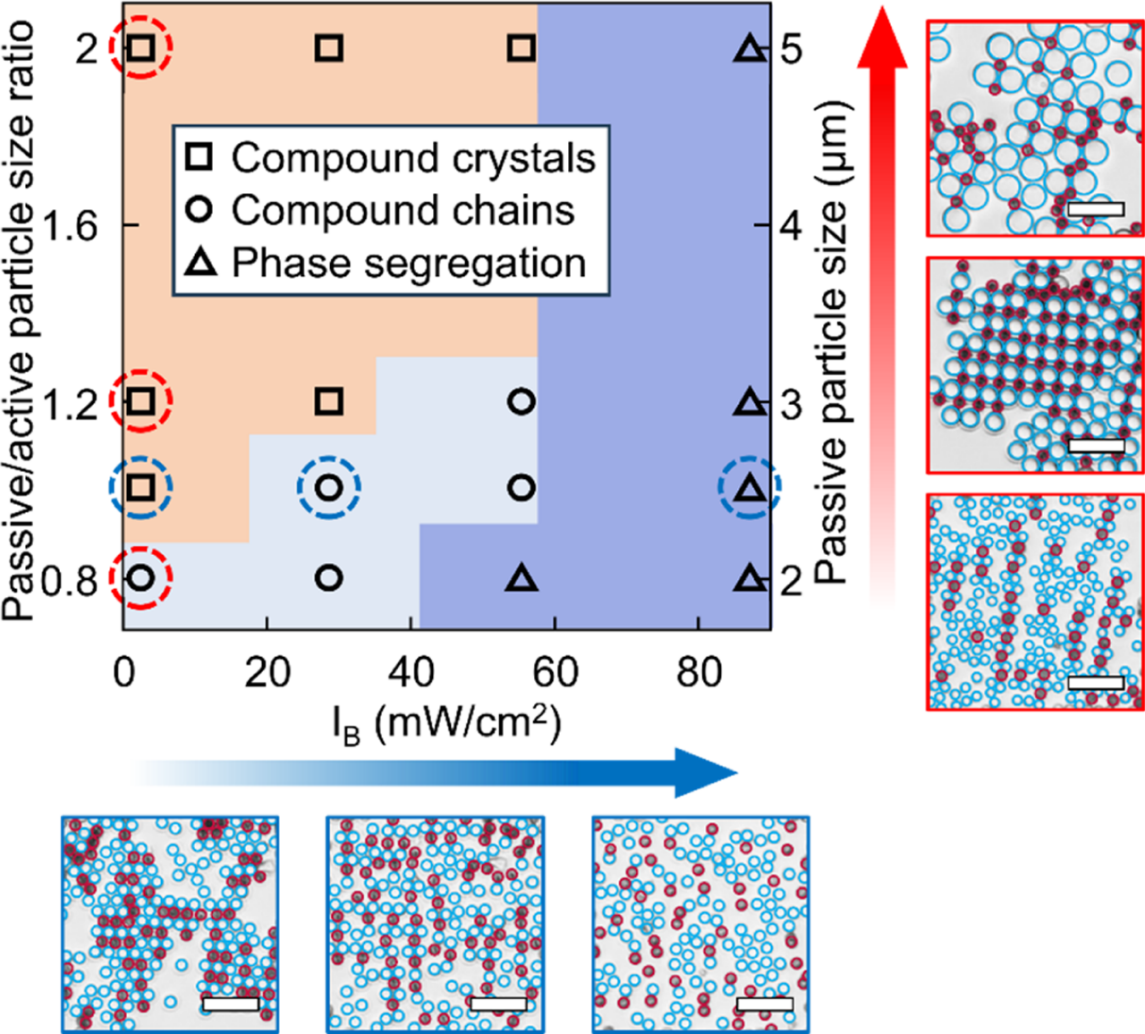
Phase diagram of binary phase formation of LEG4-TiO_2_ active particles and SiO_2_ passive particles with different sizes under different blue light intensities *I*_B_. The microscope images on the right and below the phase diagram correspond to the red and blue circled points in the diagram with TiO_2_ and SiO_2_ highlighted with red and blue circles, respectively. The blue arrow indicates the increase in light intensity, while the red arrow indicates the increase in particle size. Scale bars: 10 μm.

## Conclusions

Photoactive LEG4 dye-sensitized TiO_2_ colloidal particles are demonstrated as an intriguing model system to study self-assembly and phase transition, where the particle’s axially symmetric potential field can be easily tuned with the combination of bidirectional red light and global blue light. By adjusting the illumination, the photoactive colloid system demonstrates controllable phase transitions between the zigzag band phase, the colloidal chains, and the dispersed phase. A simple electrostatic-like interaction model is developed to capture the essence of the photochemical hydrodynamic interactions, allowing Langevin dynamics simulations to replicate the experimental phenomena. More importantly, with this potential field-tunable colloid as an active ingredient, the binary active colloid compound can be realized by mixing it with inert colloids with an appropriate size ratio. Finely controlling the shape and strength of particle interactions with external illumination allows photoactive phase transition between colloidal binary crystal, compound polymer, and phase segregation, showcasing the potential of using photoactive colloid to realize more exotic phases and assemblies for active functional materials. We believe that by advancing the illumination system to a holographic or digital light processing (DLP)-based platform will create a more programmable, spatially modulated interaction potential, enabling the self-assembly of more complex three-dimensional structures.

However, we acknowledge several limitations. First, the interaction potential is not fully quantified in real-time, and the current illumination setup (bidirectional red light and vertical blue light) introduces complexity in spatial control, limiting the scalability and programmability of interactions. For instance, deviations between experimental and simulated angular distributions at high blue light intensities (e.g., *I*_B_ = 87 mW/cm^2^) suggest that active fluctuations may play an unaccounted role, highlighting the need for more precise quantitative models. Second, the system’s reliance on external illumination constrains its application in environments where light penetration is challenging. Despite these limitations, we believe that advancing the illumination system to a holographic or digital light processing (DLP) platform could enable more programmable and spatially modulated interaction potentials, expanding the scope for designing complex three-dimensional structures. This work bridges active matter physics and solid-state chemistry, offering a foundation for future studies of optically encoded colloidal alloys.

## Materials and Methods

### Materials

Materials include the LEG4 dye sensitizer (3-{6-{4-[bis­(2′,4′-dibutyloxybiphenyl-4-yl)­amino-]­phenyl}-4,4-dihexyl-cyclopenta-[2,1-*b*:3,4-*b*′]­dithiophene-2-yl}-2-cyanoacrylic acid, product code DN-F05, Dyenamo AB, Stockholm, Sweden) for sensitization, ferrocene (1 mol/L in acetonitrile) for redox shuttle, and titanium­(IV) isopropoxide (AR grade) and acetone (AR grade) for TiO_2_ synthesis.

### Preparation of LEG4-TiO_2_ Microparticles

The 2.5 μm TiO_2_ microspheres are synthesized by a hydrothermal method. First, 2.54 mL of titanium isopropoxide (TTIP) is added into acetone with a certain ratio and a total volume of 33 mL, and the mixture is transferred into a 50 mL Teflon-lined autoclave, which is then heated in an oven for 12 h under 200 °C. The resulting yellowish precipitates are washed with isopropanol 3 times by resuspending and centrifuging. The same procedure is repeated in water another 3 times. To perform further purification and precrystallization, the final aqueous suspension is transferred to a Teflon-lined autoclave and heated to 160 °C for 24 h. The white precipitate is further dried in a 100 °C oven overnight and then annealed at 450 °C for 1 h in a furnace. Figure S1 shows the optical microscopic image as well as the SEM image. The polydispersity is calculated to be 0.0318.

### COMSOL Simulation

Hydrodynamic flow is simulated via the COMSOL Multiphysics package. In brief, a 3D model is constructed to simulate self-diffusiophoresis. The exact mechanism for ferrocene as a redox shuttle in acetonitrile solution remains unclear; however, we hypothesize that it operates similarly to the mechanism of HQ as a redox shuttle in aqueous solution and can thus be modeled analogously. We consider the area with the highest light absorption to be the source of both positive ions H^+^ and negative ions OH^–^, which is induced by the lens focusing effect. The division of the two reaction sites (focal points induced by bidirectional illumination) is shown in Figure S2a, where the reaction fluxes for both ions are set equal but with distinct diffusion coefficients. To establish the 3D model, three modules are incorporated: diffusion of dilute species and electrostatic and creeping flow. The diffusion of H^+^ (*D*_H_^+^ = 9.31 × 10^–9^ m^2^/s) and OH^–^ ( $D_{OH^{-}}=2.26\times 10^{-9}\;m^{2}/s$ ) created from the reaction site is numerically calculated by the first module, whereas the electric field caused by the uneven distribution of these two charged species is modeled by the second module. Finally, the tangential component of the electric field will drive the ions in the electrical double layer on the particle surface and produce an electric hydrodynamic flow, which is simulated by the third module.

For the calculation of transport of dilute species, the distribution of H^+^ and OH^–^ can be affected by diffusion, convection, and electrophoresis under the electric field, which can be resolved by the continuity equation at steady state,

1 $$\nabla \cdot J_{i}=u\nabla c_{i}-D_{i}\nabla^{2}c_{i}-\frac{z_{i}FD_{i}\nabla \cdot \left(c_{i}\nabla \varphi \right)}{RT}$$

where *J*_i_ is the flux of ion *I*, which is measured to be 6.25 × 10^–6^ mol/(m^2^·s), ***u*** is the fluidic velocity, *F* is the Faraday constant, *φ* is the electrostatic potential, *R* is the gas constant, *T* is the temperature, and *c*_i_, *D*_i_ and *z*_i_ are the concentration, the diffusion coefficient and the charge of species *i*, respectively. Figure S2b shows the simulated concentration distribution of H^+^ around the particle.

The electrostatic potential *φ* (*E* = −∇*φ*) from eq 1 can be calculated from the Poisson equation,

2 $${-\varepsilon }_{0}\varepsilon_{r}\nabla^{2}\varphi =\rho_{e}=F\left(Z_{+}c_{+}+Z_{-}c_{-}\right)$$

where *ε*_0_ is the vacuum permittivity and ε_r_ is the relative permittivity of acetonitrile. *Z*_+_ = 1 for H^+^ and *Z*_–_ = −1 for OH^–^. *ρ*_e_ is the volumetric charge density, *F* is the Faraday constant, *c*_+_ is the concentration of H^+^, and *c*_–_ is the concentration of OH^–^. Figure S2c shows the simulated electric field intensity distribution around the particle.

In a low Reynolds number system, the fluid flow outside the electric double layer is governed by the Stokes equation at steady state and the continuity equation for the incompressible fluid,

3 $$-\nabla p+\mu \nabla^{2}u=0$$

4 ∇·u=0

where *μ*, ***u***, and *p* are the dynamic viscosity of acetonitrile, velocity, and pressure, respectively. The initial values of ***u*** and *p* are zero in our simulation.

In the fluid flow module, the boundary condition of the particle surface is set to be the electroosmotic boundary, which is dominated by the tangential component of the electric field by

5 $$E_{t}=E-\left(E-n\right)\cdot n$$

where ***E***_**t**_ is the tangential component of the electric field strength *E*. The electroosmotic velocity is then governed by

6 $$u=-\frac{\varepsilon_{r}\varepsilon_{0}\zeta }{\mu }E_{t}$$

*ζ* here represents the zeta potential of the particle, which is 10.33 ± 0.70 mV for our LEG4-TiO_2_ particle. Figure S2d shows the simulated hydrodynamic flow field around the particle.

To extract the apparent potential from the hydrodynamic simulation, the hydrodynamic pressure exerted on nearby particles is first converted into a force field by Stokes’ law *F* = 6π*r*η*v*, and the apparent potential is then calculated by

7 $$U\left(r\right)=\int_{r}^{\infty }F\;dr$$

where the orthogonally arranged attraction/repulsion potential is plotted in Figure S2e.

### Langevin Dynamics Simulation

The simulation is conducted by using a custom implementation of Langevin dynamics in C++. Both active and passive particles, which are free to move, are initially distributed randomly within the simulation box. The interactions among active particles, between active and passive particles, and among passive particles are characterized by the directional potential with ε_rep_ = 1.1 kcal/mol, as well as a Lennard-Jones potential with ε = 1 kcal/mol. Collision diameters are assigned based on the respective particle sizes. Simulations are performed in the *NVT* ensemble, where the temperature of the system is maintained at the desired value. Periodic boundary conditions are applied in all directions to simulate an effectively infinite system. The simulations are confined to two dimensions, consistent with the observation of the active band phenomenon at the bottom of the experimental setup and the characteristics of the designed potential. The equations of motion are integrated using the velocity Verlet algorithm with a time step equal to 1 fs. Following an initial relaxation period of 50 ps, the particle dynamics are observed for an additional 200 ps. Each case is repeated 12 times to ensure statistical accuracy. The phase diagram extracted from the simulation is plotted in Figure S4, which shows good alignment with the experimental phase diagram. Our MD simulations use small timesteps to ensure numerical stability during integration of the Langevin equations, which is a standard practice in MD for systems. The model’s primary aim is to qualitatively relate anisotropic particle interactions to observed phase formation and explain experimental phenomenology rather than provide quantitative kinetic predictions or direct time scale mappings.

### Calculation of Angular Distribution

The angular distribution function *g*(*θ*) is calculated as follows. First, all neighboring particles are connected, and the angle between all pairs of particles is calculated (Figure S6a). Then, for each angle θ, the number of particle pairs within the interval [*θ* + d*θ*] is counted, where d*θ* is the step size and is taken to be 9. Due to symmetry, we only count the distribution from 0° to 180°. The angular distribution is plotted as Figure S6b.

## Supplementary Material

















## Notes

Interaction between active particles; formation of binary phases; Langevin dynamics simulation of different binary phases; and comparison to the dipolar interaction system (PDF)
Particle motion under different blue light intensities (AVI)
Phase behavior under different blue light intensities (AVI)
Orientational order parameter ψ_2_^j^ under different blue light intensities (AVI)
Phase transition by increasing blue light intensity (AVI)
Langevin dynamics simulation under different repulsion-attraction ratios (AVI)
Binary phase formation between LEG4-TiO_2_ particles and SiO_2_ particles (AVI)
Binary phase transition upon increasing blue light intensity (AVI)
