# Supplementary material for: Active Colloid Phase Transitions and Living Binary Crystal Formation
Source: ACS Nano. 2026 Feb 4;20(6):5091–101. doi: 10.1021/acsnano.5c19183 (PMC12918714; doi:10.1021/acsnano.5c19183)
Supplement: Supplementary file 1 [file nn5c19183_si_001.pdf]

Active Colloid Phase Transitions and Living Binary Crystal Formation

Jingyuan Chen#,[1,4] Shaobin Zhuo#,[2] Binglin Zeng#,[1] Zhigang Li*[2] and Jinyao Tang*[1,3,4]

[1]Department of Chemistry, The University of Hong Kong, Pokfulam, Hong Kong, China

[2]Department of Mechanical and Aerospace Engineering, the Hong Kong University of Science and Technology, Clear Water Bay, Kowloon, Hong Kong, China

[3]State Key Laboratory of Synthetic Chemistry, The University of Hong Kong, Kowloon, Hong Kong, China

[4]Materials Innovation Institute for Life Sciences and Energy (MILES), HKU-SIRI, Shenzhen, China

Supporting Notes:

Note S1. Interaction between active particles

Note S2. Formation of binary phases

Note S3. Langevin dynamics simulation of different binary phases

Note S4. Comparison to dipolar interaction system

Supporting Figures:

Figure S1. The optical microscopic image and the SEM image of the synthesized 2.5 μm TiO_2 particles

Figure S2. COMSOL simulation of hydrodynamic flow

Figure S3. Phase behavior of the LEG4-TiO_2 particles under different illumination conditions and areal density of particles

Figure S4. Phase diagram extracted from Langevin dynamics simulation

Figure S5. Interaction between active particles

Figure S6. Angular distribution of different phases

Figure S7. Formation of binary crystal

Figure S8. Langevin dynamics simulation of the binary crystals

Supporting Videos:

Movie S1: Particle motion under different blue light intensities

Movie S2: Phase behavior under different blue light intensities

Movie S3: Orientational order parameter ψ_2^j under different blue light intensities

Movie S4: Phase transition by increasing blue light intensity

Movie S5: Langevin dynamics simulation under different repulsion-attraction ratios

Movie S6: Binary phase formation between LEG4-TiO_2 particles and SiO_2 particles

Movie S7: Binary phase transition upon increasing blue light intensity

Supporting Notes

Note S1. Interaction between active particles

As shown in **Fig. S5a**, when two particles approach each other, they initially move towards one another along the illumination direction and accelerate as they get closer. Upon collision, they quickly get deflected and move in the opposite directions horizontally. As shown in the potential distribution in **Fig. S5b**, the surface along the tilted band is nearly an equipotential surface. When a new particle approaches the vicinity of the tilted band, the low potential energy at the tilted band surface attracts the particle towards the band. Subsequently, the particle undergoes a translation that closely follows the stagnation line around the active band, resulting in the observed circulating motion. We also analyze the vorticity $\omega_{z,max} = \max(|\partial v_y/\partial x - \partial v_x/\partial y|)$ for the circulating zigzag band phase. Despite the fact that the circulation of the system does not show chirality in general, the zigzag band is still locally symmetry-broken, allowing the band to form a positive-negative-positive vorticity arrangement. The simulation (**Fig. S5c-d**) well aligns with the experimental results (**Fig. S5e-f**).

Note S2. Formation of binary phases

By numerically simulating different scenarios with COMSOL, we can explain why the active particles forms a binary alloy phase with the passive particles. First, for the case of an active particle attracting one or two particles, the symmetry of its flow field is suddenly broken, which leads to a rapid movement towards the active particle end (**Fig. S7a-b**). Such enhanced motion increases the probability for the particle assembly to encounter other particles. When it encounters an active particle, it will be repelled for the aforementioned reasons. Instead, when it encounters a passive particle, the latter will be attracted to form a lager assembly. The simulation in **Fig. S7c-d** shows that the system reaches the most stable state when four passive particles are adsorbed. Additionally, the screening of the flow field becomes more pronounced when the passive particles are larger, which facilitates the formation of solid crystal.

Note S3. Langevin dynamics simulation of different binary phases

We have also performed Langevin dynamics simulations for the binary phases. For the passive particle, only a short-range repulsive potential is assigned to avoid collisions. **Fig. S8a-d** shows the simulation results for 2.5 μm active particle with 2 μm, 2.5 μm, 3 μm and 5 μm passive particles, which align well with the experiments.

Note S4. Comparison to dipolar interaction system

The zigzag band structures observed in our system are similar to those formed by dipolar particles under alternating fields[1,2]. However, the underlying interaction and the resulting system dynamics are fundamentally different. To summarize, while the zigzag patterns is superficially similar, our photoactive colloid system is distinct from dipolar systems. It is characterized by its (1) non-conservative, hydrodynamically mediated interactions; (2) exceptional optical tunability of the potential field shape; and (3) the capability for programmable multi-component "reactions".

First, the physical origin is fundamentally different. For the dipolar particles (e.g., magnetic colloids), the interaction stems from conservative dipole-dipole forces between permanent or induced magnetic moments. The potential field is static and can be directly calculated from the relative positions and orientations of the dipoles. On the other hand, for our photoactive colloids, the interaction is mediated by a non-conservative hydrodynamic flow field generated by surface photochemical reactions. This is a dissipative, solvent-mediated interaction. As visualized using tracer particles in **Fig. 1b**, the active particle creates a specific flow pattern. Although we construct an effective potential for simulation purposes (**Fig. 4a**), it is an approximation of a fundamentally hydrodynamic and non-equilibrium process.

Second, the tunability and symmetry of the interaction potential is different. For dipolar particles, the interaction strength and symmetry are largely fixed by the particle's magnetic moment and the applied field. Changing the assembly typically requires altering the global field direction or frequency. For our photoactive colloids, the shape, range, and anisotropy of the interaction potential can be dynamically and independently tuned in real-time using two wavelengths of light (IR and IB). As shown in **Fig. 1d**, increasing IB continuously reshapes the potential, reducing the attraction range along the illumination axis while enhancing orthogonal repulsion. This optical control over the potential landscape's symmetry is a key advantage over static dipolar interactions.

Finally, the extensibility to multi-component " reactions". The most significant distinction is the ability of our system to actively form stoichiometric binary "compounds" by mixing active and passive colloids. This has not yet been realized for dipolar systems, where a dipolar particle cannot attract another “inert” particle via dipolar interaction. In our system, such attraction can be realized due to the hydrodynamic nature of the interaction. This programmable, chemistry-like interaction is a unique feature of our photochemically mediated platform.

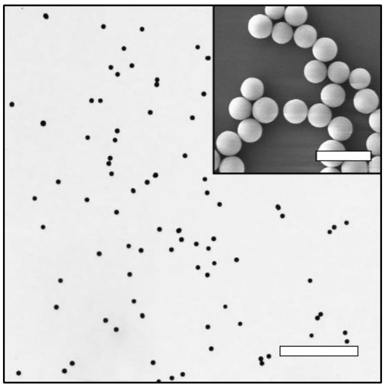

Figure S1. The optical microscopic image and the SEM image of the synthesized 2.5 μm TiO_2 particles.

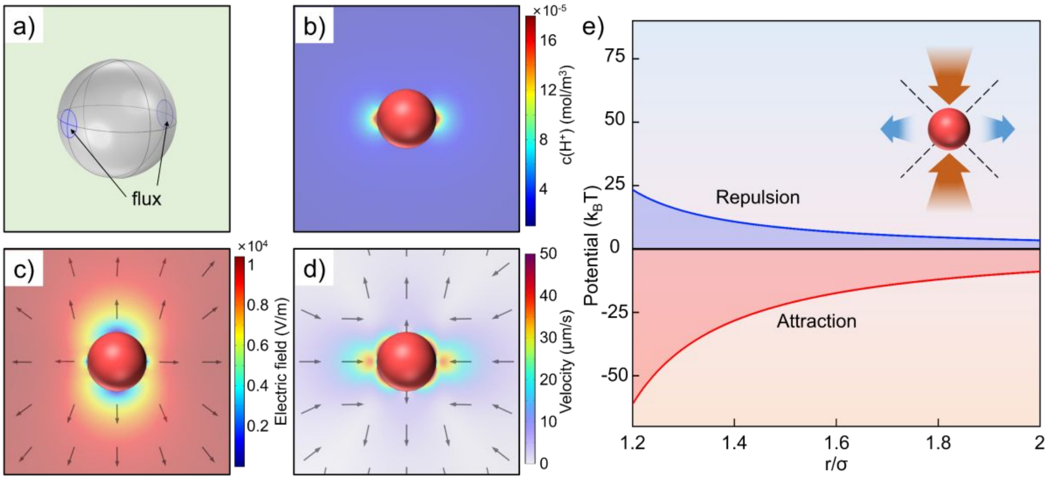

Figure S2. COMSOL simulation of hydrodynamic flow. a, Division of the bidirectional attraction site. **b**, Concentration distribution of H^+ around active particle. **c,** Electric field strength around active particle. **d,** Hydrodynamic flow around active particle. **e,** Potential curve calculated from COMSOL simulation.

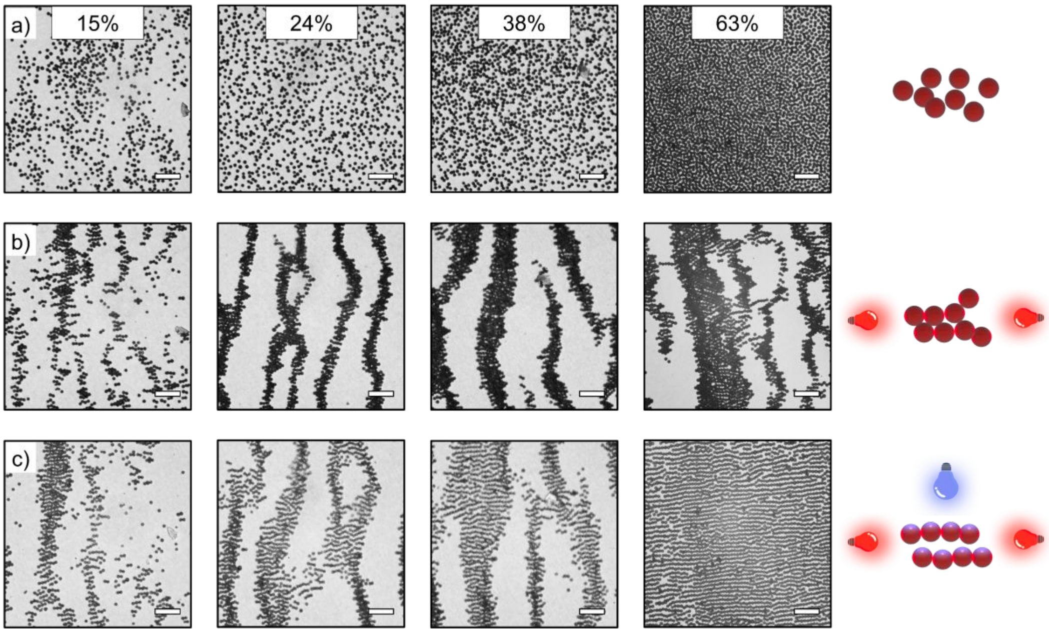

Figure S3. Phase behavior of the LEG4-TiO_2 particles under different illumination conditions and areal density of particles. a, Without illumination. **b,** With bidirectional red-light illumination. **c,** With bidirectional red-light illumination and omnidirectional blue-light illumination. Scale bars: **(a)-(c)** 10 μm.

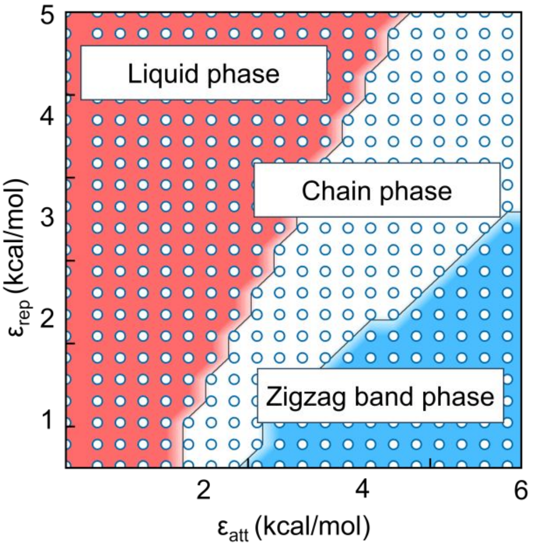

Figure S4. Phase diagram extracted from Langevin dynamics simulation.

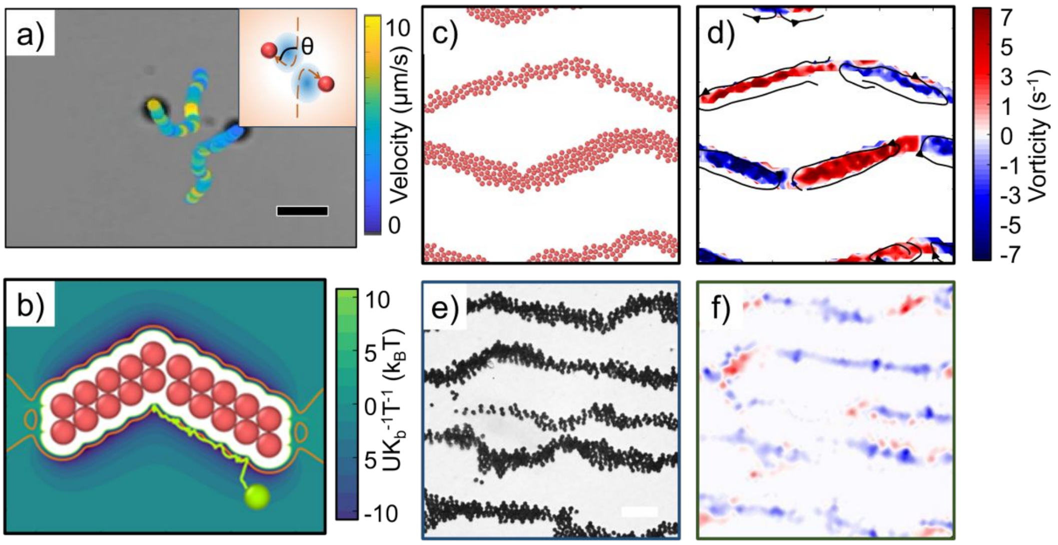

Figure S5. Interaction between active particles. a, Trajectory of two active particles approaching each other. Insert shows the particle collision angle. **b,** The trajectory of a freely moving particle (green particle) along a fixed triangular shaped band (red particles). The stagnation lines are highlighted in orange lines. **c-d,** Simulation result and the vorticity distribution on the zigzag bands showing a "+-+-" distribution. **e-f,** Experimental result and the vorticity distribution extracted from the video.

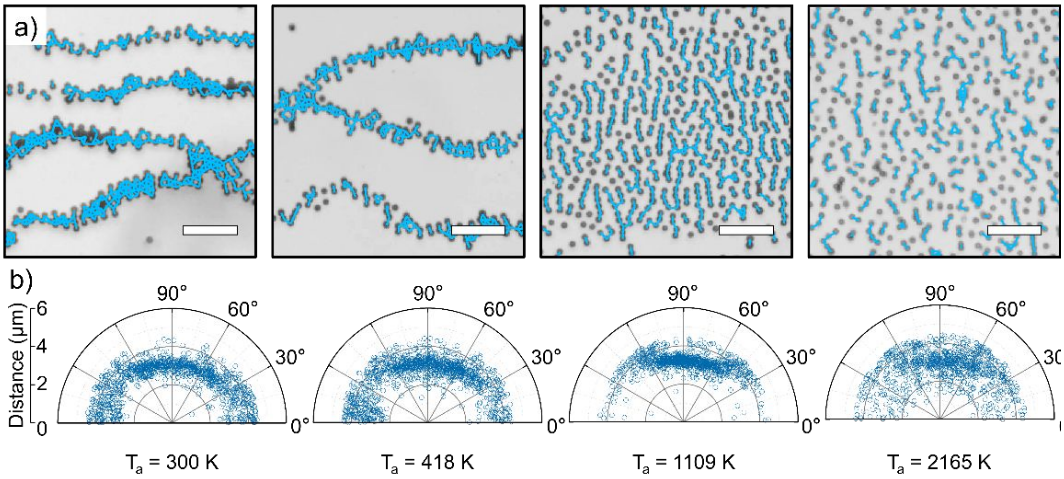

Figure S6. Angular distribution of different phases. a, Lines connecting neighboring particles for the counting of angular distribution. **b,** Angular distribution of neighboring particles.

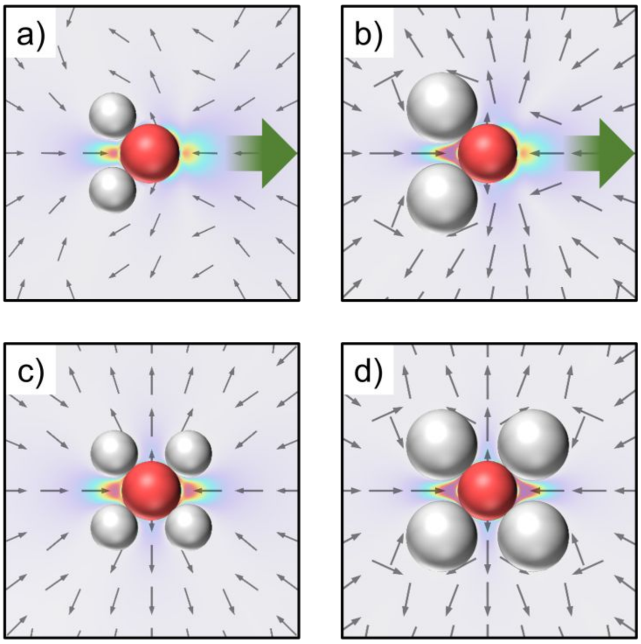

Figure S7. Formation of binary crystal. a-b, Directional motion of the particle assembly when an active particle attracts two passive particles. **c-d,** Balanced hydrodynamic flow around the particle assembly with one active particle at the center and four passive particles around.

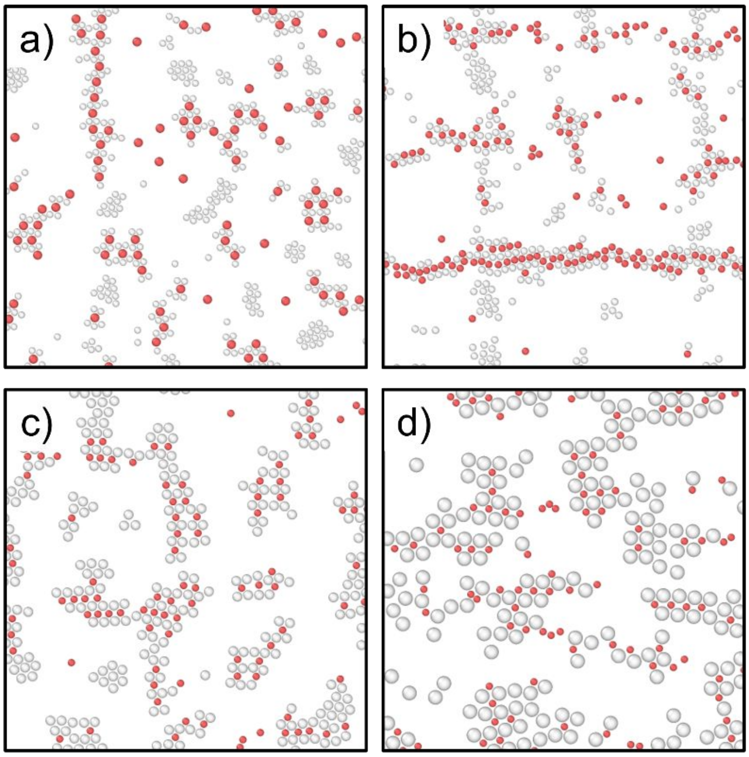

Figure S8. Langevin dynamics simulation of the binary crystals. a, With 2 μm passive particle. **b,** With 2.5 μm passive particle. **c,** With 3 μm passive particle. **d,** With 5 μm passive particle.

1 Junot, G.; De Corato, M.; Tierno, P. Large Scale Zigzag Pattern Emerging from Circulating Active Shakers. *Phys. Rev. Lett.* **131**, 068301 (2023).

2 Katzmeier, F.; Altaner, B.; List, J., Gerland, U.; Simmel, F. C. Emergence of Colloidal Patterns in AC Electric Fields. *Phys. Rev. Lett.* **128**, 058002 (2022).
